# The role of TEMRA cell-mediated immune senescence in the development and treatment of HIV disease

**DOI:** 10.3389/fimmu.2023.1284293

**Published:** 2023-10-12

**Authors:** Lihui Guo, Xudong Liu, Xin Su

**Affiliations:** ^1^ Department of Burns and Plastic Surgery, Yanbian University Hospital, Yanji, China; ^2^ Department of Rheumatology and Immunology, The First Hospital of China Medical University, China Medical University, Shen Yang, China

**Keywords:** TEMRA cells, HIV, immunosenescence, antiretroviral therapy, vaccines

## Abstract

Human Immunodeficiency Virus (HIV) has plagued human society for a long time since its discovery, causing a large number of patients to suffer and costing hundreds of millions of medical services every year. Scientists have found that HIV and antiretroviral therapy accelerate immune aging by inducing mitochondrial dysfunction, and that terminal effector memory T cells (TEMRA cells) are crucial in immune aging. This specific subset of effector memory T cells has terminally differentiated properties and exhibits high cytotoxicity and proinflammatory capacity. We therefore explored and described the interplay between exhaustion features, essential markers, functions, and signaling pathways from previous studies on HIV, antiretroviral therapy, immune senescence, and TEMRA cells. Their remarkable antiviral capacity is then highlighted by elucidating phenotypic changes in TEMRA cells during HIV infection, describing changes in TEMRA cells before, during, and after antiretroviral therapy and other drug treatments. Their critical role in complications and cytomegalovirus (CMV)-HIV superinfection is highlighted. These studies demonstrate that TEMRA cells play a key role in the antiviral response and immune senescence during HIV infection. Finally, we review current therapeutic strategies targeting TEMRA cells that may be clinically beneficial, highlight their potential role in HIV-1 vaccine development, and provide perspectives and predictions for related future applications.

## 1 Introduction

Human immunodeficiency virus(HIV) rapidly progresses to lethal acquired immunodeficiency syndrome (AIDS) if left untreated. However, with the advent of antiretroviral therapy, it is now possible to manage HIV infection as a chronic disease (1, 2). Despite this advancement, HIV infection disrupts the immune system’s homeostasis, leading to significant consequences (3). Long-term HIV infection-related cellular damage exposure accelerates cellular senescence, resulting in chronic inflammation and immune system failure (4, 5). Notably, inflammation has been identified as the second most influential factor, after age itself, in predicting outcomes such as survival, functional capability, and cognition (6). Additionally, antiretroviral therapy may contribute to HIV-associated inflammation and mitochondria-related aging in persons with HIV (PWH) (7–9). These factors together lead to a rise in the prevalence and morbidity of age-related comorbidities in PWH, including cancer, cardiovascular disease, metabolic illness, and neurodegenerative disorders (7, 10). Among those older than 40, the frequency of HIV infections has been rising quickly. According to a longitudinal study of an INDEPTH community in South Africa conducted in 2010, HIV prevalence in those 40 years and older was 21% and increased to 23% 5 years later (11). By 2030, the median age of PWH receiving combination antiretroviral therapy (ART) will be 56.6, up from 43.9 in 2010,thepercentage of PWH aged 50 years or older will be 73%, up from 28% in 2010. according to data from the Dutch AIDS Therapy Evaluation in the Netherlands (ATHENA) cohort (12, 13). Consequently, it is of utmost importance to further investigate the connection between HIV, antiretroviral therapy, and immunosenescence.

HIVand antiretroviral therapy accelerate immune senescence by inducing mitochondrial dysfunction (14, 15). Terminal effector memory T cells (TEMRA cells) are crucial in immune senescence. There are four subpopulations of T cells based on their expression of CD45RA and CCR7: effector memory T cells(TEM, CD45RA-/CCR7-), Naive T cells(TN, CD45RA+/CCR7+), central memory T cells(TCM, CD45RA-/CCR7+), and effector memory T cells re-expressing CD45RA(TEMRA, CD45RA+/CCR7-) (16). TEMRA cells, which are T cells that re-express CD45RA, represent terminally differentiated effector cells associated with protracted antigen exposure. **Table 1** presents the primary markers of TEMRA cells, particularly senescent markers. These cells are considered hallmarks of immunosenescence and are characterized by a decline in proliferation potential but strong cytotoxicity and proinflammatory activity. They generate effective effector molecules including perforins, granzymes, IFN-γ, and TNF-α (27) (28, 29). As people age, the proportion of TEMRA cells progressively increases (30). These cells exhibit various characteristics of advanced differentiation, such as a low proliferative activity, high levels of DNA damage and the loss of telomerase activity (31–33). Telomere shortening and the activation of a senescent phenotype are brought on by the relative absence of telomerase activity in TEMRA cells (34). Senescence-associated secretory phenotype (SASP), a distinctive proinflammatory secretory program, is driven by enhanced senescence-associated -galactosidase (SA-Gal) activity. TEMRA cells thrive in an inflammatory milieu and may also exacerbate it by producing multiple proinflammatory molecules recognized as contributors to SASP (32, 35). SASP is regulated by p38 MAPK signaling and has a significant role in inflammation and organismal aging (36).CD8+TEMRA cells and CD4+TEMRA cells exhibit differences in specific senescence-related characteristics. CD8+ TEMRA cells demonstrate significant mitochondrial dysfunction primarily due to decreased mitochondrial mass, leading to compromised metabolic stability and impaired nutrient uptake (37). In contrast, CD4+TEMRA cells showcase a larger mitochondrial mass and absorb more lipids and glucose than CD8+ counterparts (38). Human T cell senescence is mostly determined by mitochondrial mass, and CD8+ TEMRA cells age more rapidly than CD4+ TEMRA cells due to their increased sensitivity to senescence (38, 39). Therefore, the research findings of TEMRA cells and related treatment methods seem to provide new ideas and solutions for the treatment of HIV.

**Table 1:** Summary of significant markers of TEMRA cell.

Marker	Identification	Expression	Significance	Ref.
**CD45RA**	Receptor protein tyrosine phosphatase	+	Initiates T-cell receptor signaling	(17)
**CCR7**	CC−chemokine receptor 7	–	Participate in the lymph node homing and contribute to balance immunity and tolerance	(18)
**CD57**	A 100-115 kD terminally sulfated carbohydrate epitope	+/-	Identify terminally differentiated senescent cells with reduced proliferative capacity and altered functional properties	(19)
**CD27**	TNF receptor superfamily. (TNFRSF) target receptors	–	Drive T-cell activation	(20)
**CD28**	A founding member of a subfamily of costimulatory molecules	–	Drive critical intracellular biochemical events, including unique phosphorylation and transcriptional signaling, metabolism, and the production of key cytokines, chemokines, and survival signals	(21)
**CD31**	A transmembrane glycoprotein in the diverse immunoglobulin (Ig) gene superfamily of receptors	+/-	Recruit leukocvtes into inflammatory sites and promote angiogenesis and cardiovascular development	(22)
**CD38**	Activation-inducing surface protein on T cells	+	An Immunomodulatory Molecule in Inflammation and Autoimmunity	(23)
**CD69**	Membrane-bound receptor for type II C-lectins	+	Regulate activation and metabolic of lymphocytes	(24)
**PD-1**	Programmed Cell Death Protein 1	+	Inhibiting the apoptosis of regulator T cells and active apoptosis of antigen-specific T cells	(25)
**Ki67**	A protein highly expressed in cycling cells	+	Proliferation marker for human tumor cells regulates cell cycle progression	(26)

+: posotive; -: negative; +/-: positive/negative.

Here we aim to review the correlation between HIV, antiretroviral therapy, and immune senescence, and to summarize the characteristics, function, and regulation of TEMRA cells. We comprehensively highlighted the critical role of TEMRA cells in immunosenescence under HIV infection from multiple perspectives, including their alterations, antiviral activity, changes under antiretroviral therapy and other drug therapies, age-associated complications, and cytomegalovirus (CMV)-HIV coinfection. Based on these findings, we underlined the roles of immunosenescence and TEMRA cells in treatments and vaccines for HIV.

## 2 Mechanism

### 2.1 The interaction between HIV, antiretroviral therapy, and immunosenescence

#### 2.1.1 HIV and antiretroviral therapy cause immunosenescence and influence mitochondrial function

Immunosenescence is a dynamic and multifactorial process characterized by age-associated changes in immune responses (40, 41), which results from changes in the innate and adaptive immune systems, increasing the risk of infection, decreasing the protection provided by prior vaccines, and decreasing the response to subsequent immunizations (42, 43). Thymic involution, a significant manifestation of immunosenescence during regular aging process, reduces T cell production (44). In response to this production failure, a homeostatic process of memory T cell proliferation takes place, leading to a relative decline in T cell receptor repertoire (TCR) diversity (45).

Chronic viral infections impose a permanent stress on the immune system and reinforce immunosenescence.The enduring hyper-antigenemia such as HIV, hepatitis C virus (HCV), and CMV during the progressive decline of immune system function results in chronic inflammation, ultimately accelerating immune senescence (46, 47). The accumulation of senescent cells creates an immunosuppressive state while promoting viral replication and dissemination, ultimately exacerbating disease pathogenesis and hastening the progression to AIDS (48).

To prevent AIDS progression and the progression of viremia, PWH must be treated with antiretroviral therapy for the rest of their lives (49). The antiretroviral treatment, in particular, interferes with mitochondrial function and causes senescence in a variety of cells, despite just a brief exposure (15).

**Figure 1:**
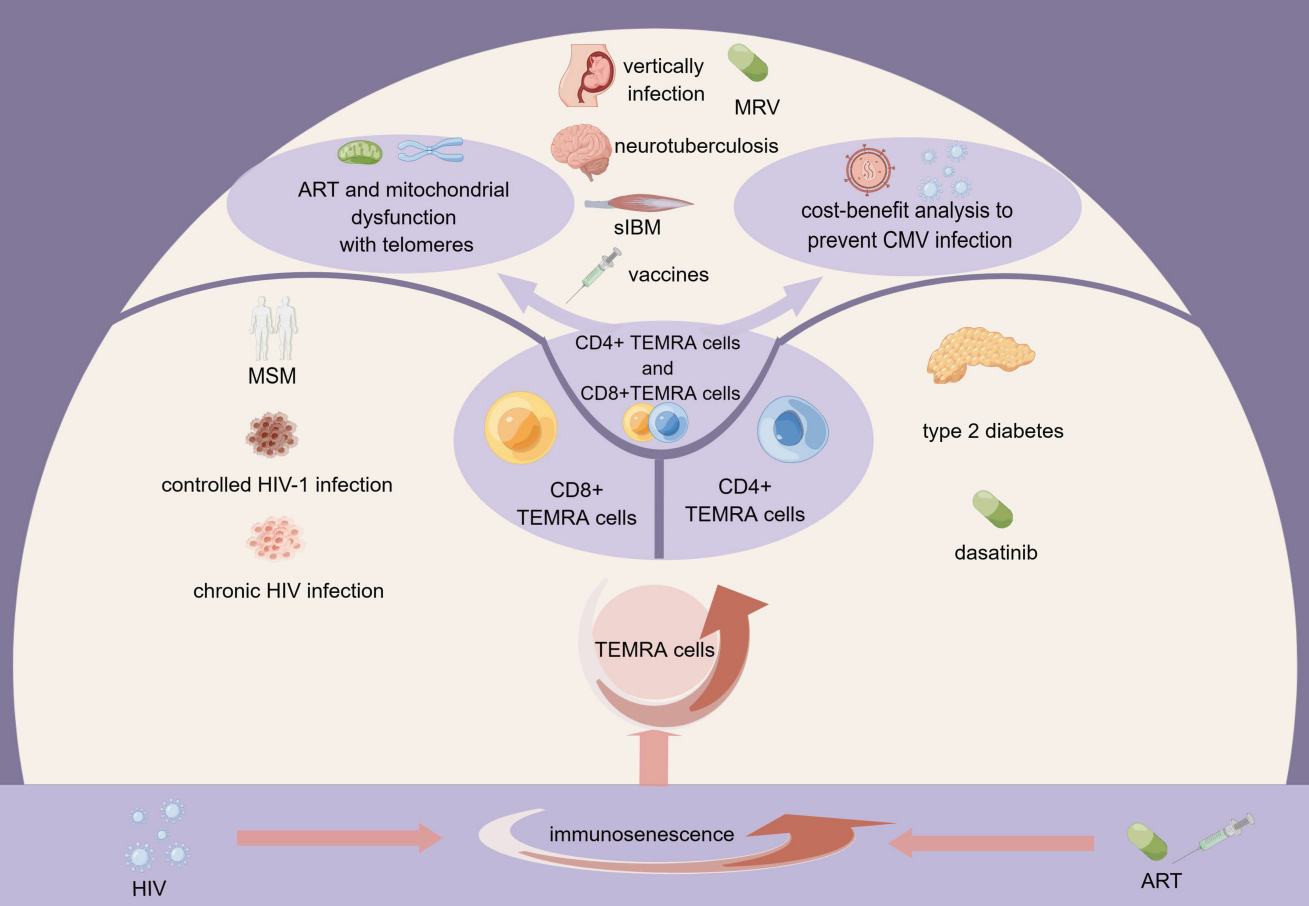
The role of TEMRA cells in HIV infection within the context of immunosenescence. HIV infection and antiretroviral therapy contribute to immunosenescence, resulting in the expansion of TEMRA cells. CD4+TEMRA cells, CD8+TEMRA or both have different connections with the clinical course of HIV infection, HIV infected population, drugs, and complications of HIV infection. The mitochondrial function of TEMRA cells and the cost-benefit analysis to prevent CMV infection are also noteworthy. MSM, men who have sex with men; ART, antiretroviral therapy; MRV, maraviroc; sIBM, sporadic inclusion body myositis; HIV, human immunodeficiency virus; CMV, cytomegalovirus; TEMRA cells, terminal effector memory T cells re-expressing CD45 RA.

Both HIV and antiretroviral therapy cause multiple impairments to mitochondrial function, including disturbances in electron transport chain (ETC) respiration and Adenosine 5’-triphosphate (ATP) synthesis, damage to mitochondrial DNA (mtDNA), disruption of mitochondrial membrane potential (▵Ψm), and increased oxidative stress. In the setting of HIV/antiretroviral treatment, increasing oxidative stress causes more mtDNA mutations. Consequently, there is a continuous loop of mtDNA damage, decreased mitochondrial function, increased oxidative stress, and reduced ATP synthesis and cellular homeostasis (50–52). Here we demonstrate the role of TEMRA cells and immune senescence from various perspectives, which will be discussed in the following sections (**Figure 1**).

#### 2.1.2 CD28 and replicative senescence

CD28 has a central role in the replicative senescence program, which likely represents a characteristic end-stage state of TEMRA cells (53, 54). According to a previous study that involved individuals from various age groups, most CD8+ T cells express CD28 at birth, where the proportion of CD28- T cells gradually rises with age (55, 56).

The absence of CD28 results in upregulating p16 and p21, two vital proteins in cell cycle regulation. The proteins inhibit cyclins and cyclin-dependent kinases responsible for converting G1 to S, leading to G1 arrest and subsequent replicative senescence (57–59).In CD8+CD27− CD28− T cells, reduced phosphorylation of a serine/threonine kinase Akt (Ser473) affects the phosphorylation of human telomerase reverse transcriptase (hTERT) (60). Additionally, down-regulation of CD28 is associated with hTERT loss, resulting in reduced telomerase activity and increased telomere fragility (61).

### 2.2 Functions and regulations of TEMRA cells

TEMRA cells, particularly CD8+ TEMRA cells, secrete substantial quantities of cytotoxic factors like perforin and granzymes, displaying a significant level of cytotoxicity (27). Additionally, they can assemble into supramolecular attack particles to carry out cytotoxic functions (62). The morphological changes include shrinkage of cells, membrane blebbing, chromatin condensation, and nuclear fragmentation that result from perforin’s disruption of intracellular endosomal membranes (63–65).

TEMRA cells’ cytotoxic potential is regulated by transcription factors T-bet and (eomesodermin)Eomes, which produce granzyme B, granzyme H, and perforin (66). Additionally, T-bet and Eomes are regulated by mammalian target of rapamycin (mTOR), a vital cellular metabolism regulator crucial in developing CD8+ T cells (67).

**Figure 2:**
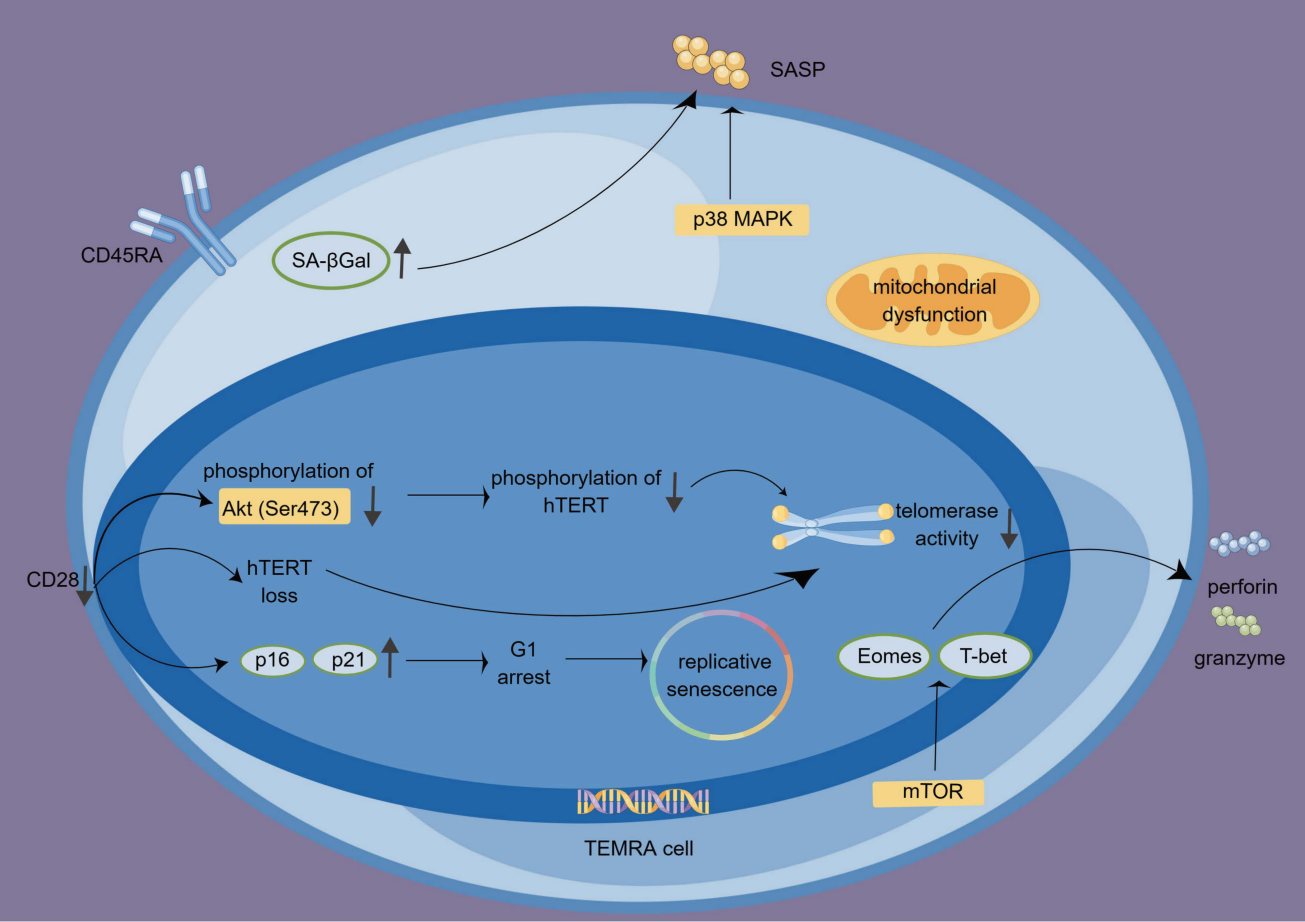
The features, functions, and regulations of TEMRA cells. The absence of CD28 influences the cell cycle, particularly G1 by upregulating p16 and p21, leading to replicative senescence. Additionally, the lack of CD28 contributes to the loss of hTERT and reduced telomerase activity. TEMRA cells produce granzyme and perforin and exhibit high cytotoxicity. This process is regulated by T-bet, Eomes, and mTOR signaling. P38 MAPK signaling participates in the SA-Gal-driven SASP. hTERT, human telomerase reverse transcriptase; SASP, senescence-associated secretory phenotype; TEMRA cells, terminal effector memory T cells re-expressing CD45 RA; SA-βGal, senescence-associated β-galactosidase.

TEMRA cells not only possess cytotoxic functions but also display a proinflammatory phenotype and secrete multiple inflammatory factors, such as interferon-gamma (IFN-γ), tumor necrosis factor-alpha (TNF-α), IL-1β, and IL-6 (28). While TEMRA cells initially produce abundant IFN-γ, their ability to do so gradually diminishes upon TCR stimulation. Nonetheless, IL-15 administration can restore their capacity for IFN-γ production. The exact mechanism underlying this restoration remains unknown but is believed to involve the mTOR pathway (68, 69). We summarize the biological characteristics, functions, and signaling pathways of TEMRA cells in **Figure 2**.

### 2.3 Interaction of TEMRA cells and HIV

#### 2.3.1 The alteration of TEMRA cells among PWH

##### 2.3.1.1 Major biomarkers alteration

Several previous studies have demonstrated alterations in TEMRA cells in PWH and healthy individuals. There is an increase in the number of TEMRA cells among PWH, as well as an increase in the expression of PD-1, CD38, CD57, and Ki67 (70). Additionally, there is an increase in the number of CD4+TEMRA cells expressing PD-L1 in the viremic HIV-1+ group (71). A PD-1 molecule or PD-L1 ligand acts as a negative regulator of T-cell activity. Furthermore, CD31 expression on memory CD8+ T cells during HIV-1 infection appears to be associated with PD-1 expression on CD8+ TEMRA cells (72). CD57 has previously been proposed as a marker of proliferative history, and more recently as a marker of HIV-specific cytotoxic CD8+ T cells, and correlated with viral suppression (73). Individuals with controlled HIV-1 infection exhibit a higher likelihood of possessing antigen-specific CD8+ TEMRA cells compared to those with progressing infection (74). High expression of EOMES among CD57+ CD8+ TEMRA cells is associated with viral control during chronic HIV infection (75). Moreover, γδT cells play a crucial role in innate immunity as the primary defense against infectious diseases (76). In individuals with acute HIV and rapid progressors, memory Vδ (2) γδ T cells display a preference for the TEMRA Vδ (2) γδ T cell phenotype. The frequency of TEMRA Vδ (2) γδ T cells is positively correlated with the frequency of CD38+ T cells, indicating that HIV infection leads to an excessive activation of TEMRA Vδ (2) γδ T cells (77). **Table 2** provides a summary of the significant TEMRA subsets that have experienced alterations, along with their respective characteristics and significance.

**Table 2:** Summary of the significant TEMRA subsets, their characteristics and significance.

Subsets of TEMRA cells	Features	Potential significance and related factors	ART treated or untreated
**CD8+ TEMRA cells**	Cytotoxic, long half-life	Accumulate more frequently in persons with controlled HIV-1 infection	untreated
**PD1+ CD8+TEMRA cells**	Negative regulator of T-cell activity	Related to CD31 expression on memory CD8+ T cells during HIV-1 infection	untreated
**PD-L1+ CD4+TEMRA cells**	Negative regulator of T-cell activity	Accumulate in the aviraemic HIV-1+ group, affect T cell functionality, especially the specific ability to generate responses against HIV-1	treated
**CD57+ CD8+TEMRA cells**	Cytotoxic (terminally differentiated feature) and poorly proliferative (replicative senescent)	Correlated with viral suppression during HIV infection	treated
**EOMES^high^CD57+CD8+ TEMRA cells**	Cytotoxic, preserve proliferative capacity and interleukin 7 (IL-7) receptor expression	Correlated with viral control during chronic HIV infection	untreated
TEMRA Vδ(2) γδ T cells	Mostly CD4- and resistant to HIV infection	Explain the dysfunction of Vδ(2) γδ T cells during acute and fast progressive HIV infection	both

##### 2.3.1.2 The senescent and activated phenotype of significant infection populations

Male homosexuality and mother-to-child transmission of HIV pose significant global health challenges (78, 79). Additionally, PWH show a statistically significant increased frequency of CD8+ memory T cell subsets with a more activated phenotype (80). In children with vertically acquired HIV-1 infection and a detectable viral load, there is an observed increase in CD8+ TEMRA cells compared to the age-matched healthy group. These CD8+ TEMRA cells also exhibit a more senescent and activated phenotype (81).. Additionally, vertically infected children have significantly higher levels of CD4+ TEMRA cells (82). These findings suggest that HIV infection induces immune activation and drives cells toward terminal differentiation, ultimately resulting in the exhaustion and depletion of these cells.

#### 2.3.2 Antiviral activity of TEMRA cells

CD8+ TEMRA cells have a strong antiviral effect. It has been shown that CD8+ TEMRA cells with HIV-1 specificity are associated with HIV-1 viremia control and can predict the set point of viral loads to come (83). Numerous studies have shown that CD8 T cells, rather than CD4 T cells, exhibit stronger activation in HIV infection with increased viremia (84). The use of consensus HIV-1 gag peptides before in vitro stimulation may increase CD8+ T cells’ viral suppressive capacity (VSC) in people with progressive HIV infection. Significantly, this enhanced VSC is correlated with greater levels of IFN-γ, TNF-α, and IL-10 production, CD8+ TEMRA cells also express immunological checkpoint markers, which are linked to T-cell exhaustion and the loss of T-cell effector functions in the context of sustained antigen exposure. In comparison to nonsuppressors, suppressor individuals have a greater fraction of PD1+CD160+CD8+ TEMRA cells, which may indicate the existence of more active and cytolytic cells that may inhibit viral replication (85). Despite prolonged viral suppression, HIV-1-infected patients are affected by impaired restoration of CD4+ T cells. In patients who demonstrate a good virological response to antiretroviral therapy, there is an association between insufficient CD4 T-cell numbers and altered CD8 T-cell responses, such as poor differentiation and fewer CD8+TEMRA cells, especially in response to Gag p24 stimulation in vitro (86). This impaired differentiation of CD8+ TEMRA cells reflects characteristics of progressive HIV-1 infection. Low CD4+ Tcell counts that continue to exist are expected to affect CD8+ TEM cell development, producing insufficient and defective CD8 +TEMRA cells that are specific for HIV-1.

#### 2.3.3 TEMRA cells in PWH and the selection of HIV-resistant CD4+ T cells

Despite their remarkable resistance to CCR5 (R5)-tropic HIV-1 infection, TEMRA cells remain highly susceptible to CXCR4 (X4)-tropic HIV-1 infection (70). It helps to understand how these individuals may sustain long-term life despite chronically low CD4+ T-cell numbers because R5-tropic virus resistance in TEMRA cells begins after viral entrance but before early viral reverse transcription. In a subgroup of HIV-infected people, a substantial positive connection between the percentage of TEMRA cells and CD4+T cell counts has also been discovered (70). Based on these findings, the development of new anti-HIV-1 treatment approaches might be aided by selecting an HIV-1-resistant CD4+T cell population.

#### 2.3.4 The changes in TEMRA cells during antiretroviral therapy

A latent viral reservoir, predominantly in CD4+ T cells, is the main reason antiretroviral therapy fails to eliminate HIV-1 in infected individuals. The majority of those treated for acute or early HIV-1 infection and HIV-1 controllers have smaller viral reservoirs. People with 50 copies of HIV-DNA per 10^6^ peripheral blood mononuclear cells (PBMCs) were shown to have a reduced percentage of CD8+ TEMRA cells in the absence of antiretroviral treatment (87). In viraemic patients, the initiation of antiretroviral therapy result in increased proportions of CD57- TEMRA cells (88).

During antiretroviral therapy, CD127– T cells were reduced within the TEMRA subset (88). School-age children, teenagers, and young adults in the virological failure (VF) group had more CD4+ TEMRA cells than those in the low-level viremia (LLV) and virological suppression (VS) groups (89).

After antiretroviral therapy, HIV-1 DNA levels and cell associated unspliced RNA (CA usRNA) levels were negatively correlated with CD8+ CCL4-CCL5+ TEMRA cells (90). Among antiretroviral therapy responders and controllers, there was a high percentage of TEMRA cells in CD4+ T cells (91). Compared to healthy controls, viral non-controllers (VNC) had a higher absolute number of CD57+ TEMRA cells, but CD57–TEMRA cells decreased after nine months of antiretroviral therapy (88), which highlighted the importance of TEMRA cells in restraining HIV-1 virus reservoirs individuals undergoing antiretroviral therapy.

These results add to a small body of evidence that the increased cytotoxic TEMRA cells have positive antiviral effects during HIV infection and antiretroviral therapy.

## 3 Clinical treatment and related applications

### 3.1 The changes in TEMRA cells under different drugs

Maraviroc (MRV) is the first CCR5 antagonist that has been approved for the treatment of HIV infection and serves as the initial antiretroviral Medications (ARVs)targeting an endogenous chemokine receptor instead of HIV itself (92). In patients with R5 multi-resistant viruses, MRV demonstrates virological effectiveness when combined with other ARVs (93). In PWH receiving eight days of MRV monotherapy, there was a notable rise in CD8+TEMRA cells and a decrease in CD4+TEMRA cells (94). The opposite effect observed in the CD4+TEMRA cells and CD8+ TEMRA cells is likely due to the blockade of CCR5. CCR5 is a receptor expressed on the surface of CD4 +T cells and is involved in various functions, including activation, migration, and survival (95). CCR5 acts as a co-receptor for certain strains of HIV-1, allowing the virus to enter and infect CD4 T cells (96, 97). In addition to its role in viral entry, CCR5 is also involved in the activation of CD4 T cells, promoting their proliferation and cytokine production (98). Further research is needed to investigate this topic. Dasatinib, a tyrosine kinase inhibitor primarily used for the treatment of chronic myeloid leukemia (CML), has been found to have an impact on HIV-1 production in vitro. Several studies have demonstrated that dasatinib can significantly block HIV-1 production in HIV-1-infected primary CD4 +T cells (99–101). Dasatinib hinders TCR-mediated activation of CD4+ T cells and obstructs the integration and reactivation of HIV-1 provirus (101). Additionally, dasatinib can preserve its antiviral function by inhibiting the phosphorylation of SAMHD1 at T592, thus having a crucial role in limiting HIV-1 replication in CD4+ T cells (102). Dasatinib also inhibits IL-2- and IL-7-induced proliferation of CD4+ T cells (103). In PWH receiving antiretroviral therapy and dasatinib, treatment with dasatinib resulted in an average 3.3-fold decrease in the proportion of CD4+ TEMRA cells compared to those receiving solely antiretroviral therapy (104). These findings underscore the significance of CD8+ TEMRA cells in limiting the reservoir in PWH undergoing antiretroviral therapy.

### 3.2 TEMRA cells’ roles in complications of HIV

PWH are prone to develop various comorbidities resulting from immune deficiency, such as Type 2 diabetes, neurotuberculosis, and sporadic inclusion body myositis (sIBM). Even though PWH may live for decades on effective antiretroviral medication, this success is offset by the population’s rising burden of metabolic illnesses (105–107). A longitudinal study carried out from 2005 to 2007 revealed a correlation between an elevated presence of CD4+TEMRA cells in peripheral blood mononuclear cells (PBMC) and the onset of diabetes in PWH (108). Furthermore, subcutaneous adipose tissue (SAT) of PWH is also enriched with CD4+ TEMRA cells, exhibiting higher CD69 expression and co-expressing CD57, CX3CR1, and GPR56 associated with increased glucose intolerance. The CX3CR1 and GPR56 markers’ expression raises the possibility that TEMRA cells have antiviral selectivity. The adipose tissue of PWH could potentially serve as a significant source of inflammation in the presence of antigen stimulation (109). This collection of virus-specific cells may contribute to adipose tissue inflammation and perhaps increase the susceptibility to developing illnesses in PWH. To determine how these cells, affect adipocytes, however, further research is required. One of the most prevalent HIV-associated opportunistic illnesses of the central nervous system in India is neurotuberculosis. HIV-TB coinfection influences the frequencies and phenotypes of CD4+ TEMRA cells and CD8+ TEMRA cells. In addition, there is a higher frequency of activated CD8+ TEMRA cells than CD4+TEMRA cells (110). sIBM has been identified as a complication of HIV/AIDS since the early days of the HIV/AIDS pandemic (111, 112). The presence of TEMRA cells is a characteristic feature of sIBM in HIV+ patients, although it is not a prerequisite for the development of IBM (113).

### 3.3 Immunosenescence and TEMRA cells in CMV coinfection populations

In populations infected with HIV, CMV) coinfection is quite common, CMV-specific CD8+TEMRA cells typically outnumber HIV-specific T cells (114). In CMV-HIV coinfection patients, the inflated CMV epitope–specific CD4+ TEMRA could potentially contribute to the higher T cell activation (115). CMV contributes to the accelerated bone loss observed in HIV disease through the proinflammatory secretory profile of CD8+ TEMRA cells (116). Moreover, CMV contributes to cardiovascular pathologies observed in HIV. Independent associations have been observed between CMV-specific T-cell responses (including TEMRA cells) and carotid intima-media thickness (117). T-cell senescence and CMV seropositivity have been identified as predictors of cardiovascular mortality, including death from myocardial infarction and stroke (118). In CMV-positive patients experiencing myocardial ischemia and reperfusion, there is a rapid loss of CD8+ TEMRA cells, potentially due to PD-1 dependent programmed cell death (119).Therefore, CMV and HIV may collectively contribute to immunosenescence, with CMV potentially exerting a more pronounced impact than HIV (120). Clinically, age-related diseases including cardiovascular disease and bone loss may get much worse as people age, which is indicative of an expedited progression of immunosenescence in chronic HIV infection.

Chronic infection with CMV contributes to the accumulation of TEMRA cells (44). CMV infection is the most common congenital infection. Recent some case reports and a small observational study show that high-dose valacyclovir may be a safe and effective preventive measure for congenital CMV (cCMV) among women with primary CMV infection in the first trimester of pregnancy (121).While prophylactic CMV immunization during infancy may be a viable strategy, It is highly unlikely that prophylactic vaccinations against CMV and HIV-1 will be developed anytime soon. There is a link between CMV and the human immune system, demanding a careful cost-benefit analysis to prevent CMV infection (122). In addition, anti-CMV viral medications usually reduce antigenic burden only when severe immunosuppression is present, such as in organ transplant recipients or individuals in advanced stages of HIV disease (123, 124).

### 3.4 Role of immunosenescence and TEMRA cells in treatments and vaccines of HIV

The buildup of CD8+ TEMRA cells, which show symptoms of replicative senescence, is a hallmark of both immunosenescence and HIV infection. Despite the positive antiviral effects of TEMRA cells, reduced numbers of these cells might lessen some of the negative clinical effects that go along with them and reduce chronic inflammation caused by TEMRA cells’ release of pro-inflammatory cytokines (124). Firstly, for the homeostatic expansion of more functional cells, it may be helpful to explore physical methods to eliminate TEMRA cells from circulation or induce apoptosis in these cells (125). As an alternative to lowering the antigenic load, CD28 or telomerase gene therapy may prevent replicative senescence and improve the activity of virus specific CD8 T cells. When CMV- or HIV-specific CD8+ TEMRA cells with intact CD28 signaling molecules were reintroduced, restoring IL-2 production and proliferative response to antigens (126). Boosting telomerase activity presents a potential strategy for preventing or delaying T lymphocyte senescence. It has been discovered that hTERT promotes enhanced proliferation, telomere length stability, and sustained antiviral action in virus-specific CD8 T cells (127).

In elderly individuals, vaccine responses might be indirectly influenced by the expansion of ‘senescence’ memory cell populations. The growing proportions of these cells within the T lymphocyte pool imply a constrained “immunological space” for the naïve repertoire, potentially contributing to the diminished response of older adults to neoantigens in vaccines (125). Nonetheless, prior research has provided evidence that the modified vaccinia Ankara-based (MVA-B) vaccine elicits targeted immune responses against the vector, partly facilitated by TEMRA cells, specifically CD8+ TEMRA cells. These responses demonstrate robust polyfunctionality and persist even after administering the third dose of MVA-B (128). These findings have significant implications for HIV-1 immunology and the broader domain of HIV-1 vaccine development.

According to the current research results, we predict the strategy and applicable direction of TEMRA for HIV treatment (**Box 1**).

**BOX 1: Future applications of TEMRA cells.**

1. As combination therapy or adjuvant therapy in ART.
2. Applied to the treatment of HIV comorbidities by elimating TEMRA cells.
3. For the development of related vaccines.
4. Treatment for improving HIV drug resistance.
5. Applied to the early diagnosis of related immunodeficiency population.

## 4 Discussion

HIV and antiretroviral therapy contribute to the acceleration of immunosenescence by inducing mitochondrial dysfunction (129, 130).These age-associated immune responses further exacerbate the inflammatory state through the expansion of TEMRA cells, and this terminally differentiated subset displays a high cytotoxic and proinflammatory phenotype. CD28 is a critical marker for TEMRA cells, exerting influence over replicative senescence by regulating the cell cycle and hTERT. To produce effective antiviral responses, TEMRA cells are essential. They also aid in the accelerated immunological senescence seen in PWH. Therefore, we emphasize that TEMRA cells have both positive antiviral effects and negative chronic inflammatory effects during HIV infection. This expansion of TEMRA cells, specifically those expressing exhaustion markers observed in PWH, suggests the involvement of T cell-mediated immune responses in the immune senescence observed during HIV-1 infection. Besides adaptive immune cells, the inflammatory microenvironment of the innate cells may also drives TEMRA cells towards senenscence non- specifically.

TEMRA cells persist in the memory T-cell pool due to their resistance to apoptosis, gradually occupying the pool and thereby restricting the repertoire of remaining T cells (131). This review revealed that the exhaustion marker CD57 can influence the anti-apoptotic properties of TEMRA cells. CD57-TEMRA cells exhibit a longer half-life, declining until nine months after antiretroviral therapy initiation. Conversely, CD57+TEMRA cells are more vulnerable to activation-induced cell death upon antigen stimulation. The presence of HIV (either as reservoirs or with detectable viral load) may induce apoptosis in the CD57+TEMRA subset.

Furthermore, the expression of immune checkpoint markers on TEMRA cells is correlated with viral suppression. The groundwork for future research into the immunological profiles linked to viral suppression is laid by the widespread recognition of these immune checkpoint markers as signs of cellular fatigue. The expression of immune checkpoint markers has been associated with T-cell exhaustion in chronic diseases characterized by prolonged antigen exposure, resulting in a progressive decline in effector functions (132). CD4+ TEMRA cells are strongly linked to more severe immunological suppression during HIV infection, whereas CD8+ TEMRA cells exhibit robust antiviral activity. Despite our identification of the accumulation and phenotypic alterations of TEMRA cells during antiretroviral therapy and specific drug therapies, additional research is necessary to ascertain the specific subsets of TEMRA cells contributing to the suppression of viral replication. Additionally, the discrepancies in TEMRA cell markers and protein levels still require clarification. Among PWH, the diverse behaviors exhibited by TEMRA cells at various stages of differentiation may have significant implications for future treatment strategies. Specific drugs, like MRV, might have distinct effects on TEMRA cells, resulting from the blockade of the CCR5 receptor. Consequently, more extensive research is needed to examine this subject in greater detail.

We believe that these alterations in TEMRA cells, which result from comorbidities of HIV infection, are specific to certain organs. Due to the modified immune status caused by HIV infection, along with notable clinical distinctions like an earlier onset of illness and potentially accelerated disease progression, it is advisable to classify patients with varying HIV comorbidities into subgroups for future investigations. These differences in cytotoxic TEMRA cell differentiation stages may be relevant for potential novel therapeutic strategies. Long-term chronic exposure to CMV plays a significant part in accelerated immunosenescence, which is defined by replicative senescence-related traits, notably the increase of CD8+ TEMRA cells. Reactivation of CMV can also lead to severe complications. Regardless of the underlying causes for the increase of TEMRA cells in PWH, lowering the percentage of these cells may lessen many of the negative clinical outcomes and lessen the severity of age-related pathologies. Further investigation is needed to explore strategies for delaying the generation of senescent CD8+ TEMRA cells. Researchers working on vaccine development have a big difficulty because of the aging immune system. Yet, new vaccine trials to induce antibodies and T-cell immunity have already been initiated, and the results will be revealed in the upcoming years.

In conclusion, the research on TEMRA cells and the exploration of immune senescence may to provide new treatment ideas and research directions for immunodeficiency patients and PWH. The application of related combination therapy and the research and development of vaccines also have huge medical market value. We believe that with the development of technology and in-depth research in related fields, we can eventually treat and prolong the survival of PWH through various means such as drugs or vaccines.

## Author contributions

LG: Writing – original draft. XL: Writing – review & editing. XS: Writing – review & editing. 
